# Non-coding RNA in drug resistance of hepatocellular carcinoma

**DOI:** 10.1042/BSR20180915

**Published:** 2018-10-10

**Authors:** Bisha Ding, Weiyang Lou, Liang Xu, Weimin Fan

**Affiliations:** 1 Program of Innovative Cancer Therapeutics, Division of Hepatobiliary and Pancreatic Surgery, Department of Surgery, First Affiliated Hospital, College of Medicine, Zhejiang University, Hangzhou 310003, China; 2 Key Laboratory of Combined Multi-Organ Transplantation, Ministry of Public Health, Hangzhou 310003, China; 3 Key Laboratory of Organ Transplantation, Zhejiang Province, Hangzhou 310003, China; 4 Department of Pathology and Laboratory Medicine, Medical University of South Carolina, Charleston, SC 29425, U.S.A.

**Keywords:** chemoresistance, drug resistance, hepatocellular carcinoma, HCC, long non-coding RNA, microRNA

## Abstract

Hepatocellular carcinoma (HCC) has been one of the most highly lethal cancers. The acquisition of drug resistance accounts for the majority of poor effects of chemotherapy in HCC. Non-coding RNAs (ncRNAs) including miRNAs, long ncRNAs (lncRNAs), and circular RNA (circRNA) have been well-documented to participate in cancer occurrence and progression. Recently, multiple studies have highlighted the key roles of ncRNAs in chemoresistance of HCC. In addition, accumulating evidence has demonstrated that they can serve as biomarkers in diagnosis, treatment, and prognosis of HCC. In this review, we first overviewed up-to-date findings regarding miRNA and lncRNA in drug resistance of HCC, then summarized specific mechanisms that they modulate chemoresistance of HCC, and finally discussed their potential clinical application in overcoming the obstacle of HCC chemoresistance in the future.

## Introduction

Hepatocellular carcinoma (HCC) is one of the most frequently diagnosed cancers and the second leading cause of cancer-related deaths amongst males worldwide, which is largely caused by chronic hepatitis B virus (HBV) infection [1]. In parts of Western countries, the mortality of HCC continues to grow and seriously affects public health [1]. In general, many treatments are curative for early HCC, such as transplantation, surgical resection, and chemotherapy [2]. However, the absence of obvious early symptoms results in most HCC cases being first diagnosed at advanced stage. The principal therapeutic agent for advanced HCC, sorafenib, is greatly limited by its drug-resistance [3,4].

Non-coding RNAs (ncRNAs) refer to RNAs that do not encode proteins. It is well recognized that ncRNA makes up a vast majority of cellular RNAs, accounting for greater than 90% of human RNAs [5]. Recent studies have shown that ncRNAs, just as important as proteins, act as underlying players in multiple cellular processes, such as cell proliferation, migration, apoptosis and angiogenesis, and immune response [6]. Non-coding variants are closely linked to most of common diseases, such as human cancers [7]. Additionally, ncRNAs are involved in drug resistance in multiple types of cancer including HCC [8]. The majority of ncRNAs involved in drug resistance are miRNAs and long ncRNAs (lncRNAs) [9]. miRNAs are a class of non-coding single stranded RNA molecules, which are constituted by approximately 22 nucleotides. Recent studies have found an association between miRNAs and drug resistance in HCC [10–14]. LncRNAs are ncRNAs with a length more than 200 nucleotides, which have been shown to interplay with multiple ‘biological elements’ including DNA, RNA, and protein [15]. Through these approaches, lncRNAs exert their effects in various physiological and pathobiological processes like autophagy and metastasis [16–18]. Additionally, lncRNAs also mediate chemoresistance of HCC, offering a new diagnostic marker and therapeutic target for HCC [19, 20]. In addition to miRNA and lncRNA, another type of non-coding RNA- circular RNA (circRNA) has been recently entered into the eyes of researches and scholars. It has been reported to act as a sponge for miRNAs, thereby participating in a series of biological and pathological processes as well as drug resistance [21]. Herein, this review summarized the relationship between ncRNAs and drug resistance of HCC.

## MiRNAs

### miRNA and multidrug resistance

#### Multidrug resistance

During the long-term of traditional chemotherapy for HCC, multidrug resistance (MDR) occurs frequently, leading to the relapse of cancer and intractable tumor [22]. Many mechanisms contribute to this resistance. One is that cancer cells enhance the ability of the efflux of hydrophobic cytotoxic drugs [23], partly through overexpression of ATP-binding cassette (ABC) transporters family, including P-glycoprotein (P-gp) and MDR-associated protein (MRP) [24–27], and decrease the uptake of hydrophilic drugs like cisplatin [28]. Recent investigations have shown that 170 kd membrane glycoprotein (170 GP) also has a close relationship with MDR [29]. Another crucial mechanistic branch leading to MDR is resistant to cell apoptosis. For example, wild-type p53 (*wt-p53*) gene re-sensitizes Bel-7402 cells to VCR chemotherapy [30]. Furthermore, *wt-p53* regulates expression of genes of enzymes to mediate function such as activation of pro-drugs, inactivation of active agents, DNA damage repair, modification of stem cells, metabolic alterations, and microenvironment change [14,23,28,31,32]. Various kinds of pathways are related to chemoresistant phenotypes in tumor such as TRPC6/calcium/STAT3 pathway in HCC [33].

#### Dysregulated miRNAs related with MDR in the treatment of HCC

##### miRNAs in HCC

*MiR-122* could not only adverse to cisplatin resistance in cisplatin-treated HepG2 cells [34], but also make HCC cells re-sensitize to adriamycin (ADM) and vincristine by down-regulating MDR related genes, such as *MDR-1, MRP, GST-pi* [35]. In another study, Wu et al. [36] have found a novel regulatory pathway (Hnf4α/miR-122/GALNT10) that could increase sensitivity of cancer cells to doxorubicin and sorafenib. *MiR-223* could decrease expression of ABCB1 at both mRNA and protein levels, which could decrease doxorubicin IC_50_ dose of HCC cells [37]. *MiR-216b* regulated MDR of HCC via mediating modification of autophagy through HIF-2α-MALAT1-*miR-216b* axis [38]. One study has found that *miR-27a* might reverse chemoresistance in HCC by inhibiting FZD7/β-catenin pathway [39]. *MiR-612* mediated the function of anti-MDR also through β-catenin pathway, and finally relieved 5-fluorouracil (5-FU) and cisplatin resistance [40]. Furthermore, *miR-34a* could also re-sensitize the effect of radiotherapy by inhibiting LDHA [41]. Zhao et al. [42] demonstrated that *miR-491*-3p/Sp3/ABCB1 axis could offer a new pathway for chemotherapy of HCC. Up-regulation of *miR-503* suppressed HCC cells proliferation, sensitized HCC cells to chemotherapeutic agents like 5-FU [43], moreover, reversed ADM and cisplatin resistance [44,45]. Besides, *miR-137* [46], *miR-205-5P* [47], and *miR-27b* [48] also have shown a low expression and close correlation with chemoresistance in HCC. Let-7 family consists of 11 closely related genes. Most of them acted as tumor suppressor like Let-7g [49]. Let-7g increased the effectiveness of fluorouracil in treating Bel-7402/5-Fu by targeting on the *HMGA2* gene. However, some Let-7 family members were up-regulated in certain tumors and promoted tumor progression, like Let-7a in HCC [50]. Besides, *miR-199a* could not only increase sensitivity to cisplatin via enhancement of autophagy by targeting autophagy-associated gene 7(*ATG7*) [51], but also increase doxorubicin sensitivity of HCC cells by regulating mammalian target of rapamycin (mTOR) and c-Met [52] (Table1).

**Table 1:** Summary of miRNAs involved in multiple drug resistance in HCC

Dysregulation	miRNA	Pathway/target	Corresponding drugs	References
**Down-regulated**	*miR-122*	MDR-1, GST-pi, MRP, Bcl-w, cyclinB; Hnf4α/*miR-122*/GALNT10 pathway	Adriamycin; vincristine; sorafenib; doxcrubicin; cisplatin	[34–36]
	*miR-223*	ABC1	Doxorubicin; paclitaxel	[37]
	*miR-216b*	HIF-2α-MALAT1-*miR-216b*	5-FU; adriamycin; cisplatin; Mitomycin C	[38]
	*miR-27a*	FZD7/β-catenin pathway	5-FU; adriamycin; Mitomycin C	[39]
	*miR-612*	Wnt/β-catenin signaling	Cisplatin; 5-FU	[40]
	*miR-491-3p*	*miR-491*-3p/Sp3/ABCB1 axis	Doxorubicin; vinblastine	[42]
	*miR-503*	EIF4E	5-FU; adriamycin; cisplatin	[43–45]
	*miR-137*	FBI-1	Adriamycin	[46]
	*miR-205-5P*	PTEN/JNK/ANXA3 pathway	5-FU	[47]
	*miR-27b*	p53; CYP1B1	Doxorubicin; sorafenib; Epirubicin	[48]
	Let-7 g	HMGA2 gene	5-FU	[49]
	*miR-199a* (3p/5p)	ATG7; mTOR and c-Met	5-FU; doxorubicin	[51, 52]
**Up-regulated**	Let-7a	Caspase-3I	Interferon-γ; doxorubicin; paclitaxel	[50]
	*miR-21*	PETN, PDCD4	Interferon-α; 5-Fu; cisplatin	[34, 53]
	*miR-183*	IDH2/SOCS6-HIF-1α	5-FU	[54]

##### Up-regulated miRNAs in HCC

*MiR-21* plays a vital role in modulating anti-tumor effect of 5-FU and interferon (IFN)-α on HCC cell lines and clinical patients with HCC [53]. The team of Wang and co-workers suggested that *miR-183* promoted MDR in HCC cells by regulating *miR-183*-IDH2/SOCS6-HIF-1α feedback loop. Both *miR-183* knockdown and SOCS6 overexpression sensitized BEL-7402/5-FU cells to 5-FU [54].

### miRNAs mediate single drug resistance of HCC

#### miRNAs and sorafenib

At present, acquisition of sorafenib resistance is a primary limitation of sorafenib-based chemotherapy. *MiR-338-3p* was proved to sensitize sorafenib in HCC by down-regulating hypoxia-induced factor 1α, which is significant for hypoxia signaling pathway [55]. Additionally, *miR-193b* increased the sensitivity of HCC cells to sorafenib [56]. *MiR-494* increased sorafenib resistance to HCC cells by targeting PTEN. [57]. *MiR-34a* was reported to increase the effect of sorafenib in HCC cells *via* direct suppression of *Bcl*-2 [58]. Besides, Xia et al. [59] found that *SMAD7* (one of the TGF-β type 1 receptor antagonists) and *PTEN* were two functional targets of *miR-216a/217*. By targeting PTEN AND SMAD7, *miR-216a/217* activated the PI3K/Akt and TGF-β pathways, thereby promoting drug resistance and recurrence of liver cancer (Table2).

**Table 2:** Summary of other miRNAs involved in single drug resistance in HCC

Drugs	miRNA	Pathway/ target	Dysregulation	References
Sorafenib	*miR-338-3p*	HIF-1 α	Down-regulated	[55]
	*miR-193b*	Mcl-1	Down-regulated	[56]
	*miR-494*	PTEN, PI3K and p-Akt	Up-regulated	[57]
	*miR-34*	Bcl-2	Sorafenib	[58]
	*miR-216a/217*	PTEN; SMAD7	Sorafenib	[59]
Cisplatin	*miR-363*	Mcl-1	Down-regulated	[60]
	*miR-182*	TP53INP1	Up-regulated	[64]
	*miR-130a*	Wnt/β-catenin	Up-regulated	[65]
	*miR-340*	Nrf2-dependent antioxidant pathway	Down-regulated	[67]
	*miR-33a-5p*	/	Down-regulated	[68]
5-FU	*miR-141*	Nrf2-dependent antioxidant pathway	Up-regulated	[69]
	*miR-195*	BCL-w	Down-regulated	[70]
	*miR-193a-3p*	SRSF2	Up-regulated	[72]
Doxorubicin	*miR-26*	ULK1	Down-regulated	[73]
Gemicitabine	*miR-106a*	PDGF-D/*miR-106a*/Twist1 pathway	Down-regulated	[74]
Adriamycin	*miR-215*	DHFR and TS	Up-regulated	[75]
	*miR-31*	NDRG3	Down-regulated	[76]
Etoposide	*miR-23a*	TOP1	Up-regulated	[77]
Radiation	*miR-20a*	PTEN/PI3K/Akt signaling pathway	Up-regulated	[78]
Interferon-α	*miR-146a*	SMAD4	Up-regulated	[79]
Arsenic trioxide	*miR-539*	Bcl-2 and Bcl-xL	Down-regulated	[80]

#### miRNAs and cisplatin

Apoptosis is a critical underlying mechanism contributing to cisplatin resistance. Recently, numerous studies have shown that miRNAs work in regulating the cisplatin resistance via targeting apoptosis-associated signaling pathways. For instance, *miR-363* reverses cisplatin resistance of HCC cell by directly targeting 3′-UTR of Mcl-1 [60]. Tumor protein p53-induced nuclear protein 1(*TP53INP1*) promotes the activity of *p53*, which has confirmed to be related with tumor cell apoptotic progression [61–63]. *MiR-182* expression was negatively correlated with *TP53INP1* both *in vitro* and *in vivo* [64]. In addition, *miR-130a* increased drug resistance [65]. Sulforaphane, one of the best anticancer plant active substances discovered in vegetables, could prevent apoptosis in BALB/c mice by activating the defensive response that mediated by NF-E2-related factor 2 (Nrf2), revealing a underlying relationship between Nrf2 and cell apoptosis [66]. Besides, *miR-340* has the ability of reversing cisplatin resistance by regulating Nrf2-dependent antioxidant pathway, supporting that *miR-340* may be a potential candidate for treating cisplatin resistance of HCC [67]. Up-regulation of *miR-33a-5p* also increased the sensitivity of HCC cells to cisplatin [68].

#### miRNAs and 5-fluorouracil

*MiR-141* reversed the resistance of HCC cells to 5-FU *via* the Nrf2-dependent antioxidant pathway [69]. Yang et al. [70] reported that overexpression of *miR-195* markedly decreased the level of anti-apoptotic protein Bcl-w, and improved the sensitivity of 5-FU in HCC. Furthermore, down-regulation of SRSF2 (a splicing factor) could induce apoptosis [71]. By repressing SRSF2, DNA methylation-regulated change in the expression of *miR-193a-3p* consequently increased the 5-FU resistance of HCC cells [72].

#### Other miRNAs involved in drug resistance in HCC

Apart from those miRNAs mentioned above, *miR-106a, miR-215, miR-23a, miR-20a, miR-146a, miR-539, miR-31, miR-26*, and *miR-33a-5p* also show underlying ability to regulate resistance during the treatment of HCC. Although *miR-26* reverses the effect to doxorubicin sensitivity [73]. In addition, inhibit the expression of *miR-106a* offered HCC patients a novel treatment strategy [74]. ADM also named adriamycin is a frequent chemotherapy medication utilized to treat multiple types of cancers. Recent study indicated that up-regulation of *miRNA-215* resulted in insensitivity to ADM by directly targeting dihydrofolate reductase (DHFR) and thymidylate synthase [75]. However, overexpression of *miR-31* exerted opposite effect [76]. *MiR-23a* could enhance the anti-tumor effect of etoposide in HCC by inhibiting topoisomerase 1 expression [77]. *MiR-20a* resensitized HCC cells to radiotherapy via PTEN/PI3K/Akt pathway [78]. *MiR-146a* regulate the effect of IFN-α to HCC cells by mediating SMAD4 [79]. Additionally, *miR-539* induced HepG2 cells apoptosis and remarkably overcame arsenic trioxide resistance [80].

## LncRNAs

In recent studies, lncRNAs are widely recognized as crucial regulators in suppressing tumor and oncogenesis, and emerge as potentially vital mediators in regulating drug resistance through modulation of apoptosis, drug efflux system, drug metabolism, DNA repair, and EMT [81–83]. A new study finds that ncRNAs including lncRNAs can participate in drug resistance mediation by controlling the function of cancer stem cells [84]. Li et al. [85] demonstrated that lncARSR was involved in doxorubicin resistance during the treatment of HCC. By knockdown of lncARSR, *PTEN* expression was decreased whereas PI3K/Akt pathway was activated. Thus, sensitized HCC cells reacted with the resistance of doxorubicin. Linc-VLDLR contributed to improve drug resistance of HCC patients by regulating chemotherapeutic agents transport, in other words, by modulating expression of drug transporter genes, like ABC subfamily G member 2 *(ABCG2*), leading the development of sorafenib-resistance, but decline the viability of cells [86]. As we all know, TGF-β is a key factor related with drug resistance of human cancers. LncRNA ribonucleic acids-ROR (lncRNA-ROR) is a functional player in chemoresistance during the treatment of chemotherapy. Recent study showed that TGFβ selectively enriched lnc-RoR within extracellular vesicles, thereby promoting HCC chemoresistance [87]. Tsang and Kwok [88] have found that knockdown of H19 by transfecting antisense H19 oligonucleotides suppressed the expression of *MDR1* gene as well as its protein product P-gp, and increased doxorubicin sensitivity in both R-HepG2 cells and HepG2 parent cells, which was partially due to the regulation of *MDR1* promoter methylation by H19. Xiong et al. [89] provided a new insight into the function of HULC/USP22/silent information regulator 1 (Sirt1)/protective autophagy pathway and demonstrated the capacity of lncRNA HULC to decrease chemosensitivity of HCC cells. To be specific, HULC up-regulated ubiquitin-specific peptidase 22 (USP22) through down-regulating three miRNAs and stabilizing Sirt1 protein. Therefore, triggered protective autophagy was harmful for patients with HCC. Taurine up-regulated gene 1 (*TUG1*) is a lncRNA that was identified to be related with tumor cells apoptosis and was up-regulated in ADM-resistant cells. Yang et al. [90] found that down-regulation of *TUG1* attenuated the resistance of HCC cells to chemotherapy via suppressing the expression of *MDR1* and P-gp. In addition, after transfected with *TUG1* siRNA and treated with ADM, SMMC-7721/ADM, and HepG2/ADM cells showed higher apoptosis rate. Furthermore, HANR, NR2F1-AS1, and HOTAIR were lncRNAs up-regulated in HCC tissue. Down-regulation of HANR enhanced chemosensitivity to doxorubicin in HCC cell lines [91], NR2F1-AS1 regulated HCC oxaliplatin resistance by targeting *miR-363-ABCC1* pathway [92], and knockdown of lncRNA HOTAIR sensitized HCC cells to cisplatin through regulating the STAT3/ABCB1 signaling pathway [93]. Besides, Schmitt et al. [94] suggested that lncRNAs can be regulated by p53. Thus, p53 modulated lncRNAs may be one of the mechanisms for drug resistance in HCC (Table 3).

**Table 3:** Dysregulated lncRNAs involved in drug resistance in HCC

LncRNA	Expression in HCC	Drug	Mechanism	References
LncARSR	Up-regulated	Doxorubicin	Modulating PTEN-PI3K/Akt pathway	[85]
LincRNA-VLDLR	Up-regulated	Sorafenib, camptothecin, doxorubicin	Reducing expression of ABCG2	[86]
LincRNA-ROR	Up-regulated	Sorafenib	Response to TGF-β	[87]
H19	Up-regulated	Doxorubicin	Inducing P-gp expression and regulating MDR1 promoter methylation	[88]
HULC	Up-regulated	Oxaliplatin, 5-fluorouracil, pirarubicin	Triggering autophagy via stabilizing Sirt1	[89]
TUG1	Up-regulated	Adriamycin	Promoting expression of P- gp and MDR1	[90]
HANR	Up-regulated	Doxorubicin	Regulating the phosphorylation of GSK3β	[91]
NR2F1-AS1	Up-regulated	Oxaliplatin	Targeting *miR-363*-ABCC1 pathway	[92]
HOTAIR	Up-regulated	Cisplatin	Activating STAT3/ABCB1 pathway	[93]

## Conclusions

**Figure 1:**
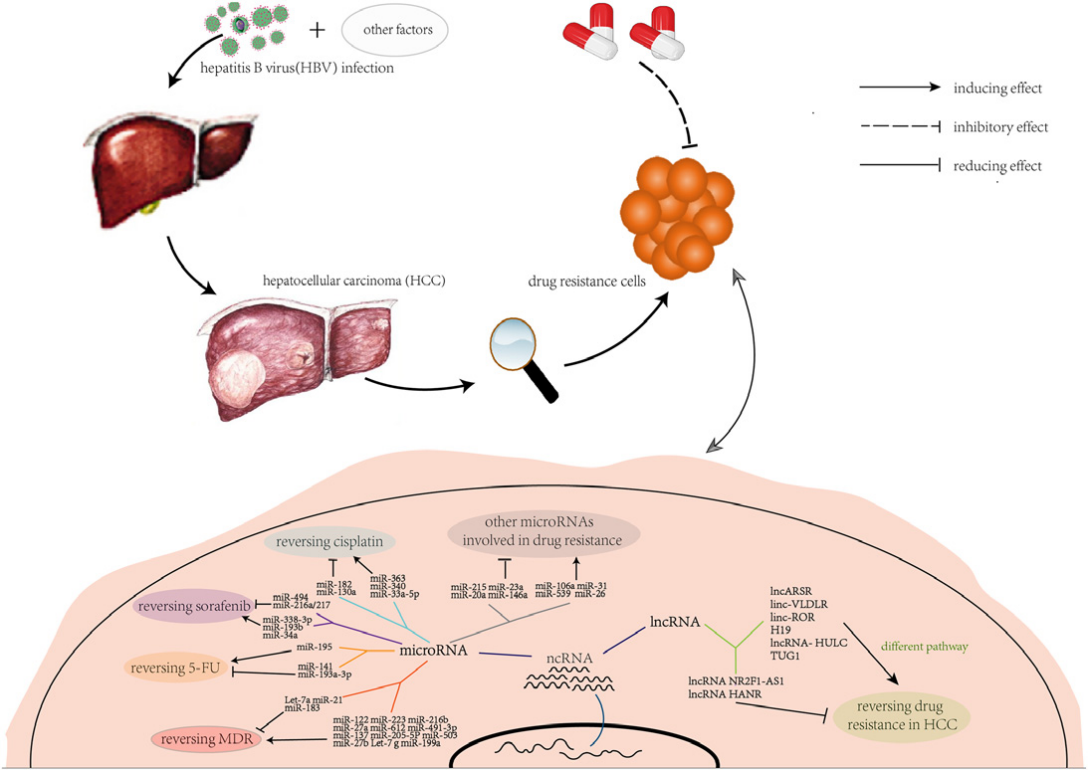
NcRNAs involved in drug resistance of HCC HCC largely caused by chronic HBV infection, and the effect of chemotherapy was seriously limited by drug resistance. A lot of ncRNAs are involved in drug resistance of HCC. These ncRNAs can mediate the sensitivity of single-antitumor drug or multi-antitumor drug of HCC, and the molecular mechanisms constitute a complicated network machinery.

Clearly, drug resistance is the one that causes the most trouble during the therapy of HCC in clinic settings and need urgent solution. NcRNAs including miRNAs, lncRNAs, and circRNAs are suggested to be the potential promising therapeutic targets for overcoming drug resistance in the treatment of HCC. Advanced experimental techniques including RNA-sequencing, CRISPR screens, genome wide association studies and high-throughput studies allow characterizing novel ncRNA roles in HCC drug resistance. Molecular mechanisms of ncRNAs in HCC constitute a complicated regulatory network (Figure 1). Although a large biological signal pathways of ncRNAs involved in drug resistance are still unknown. More mechanisms and functions of chemoresistance-related ncRNAs need to be further mined for advance of HCC therapy, which may offer new approaches to reverse drug resistance. Interestingly, this paper finds that some miRNAs belong to same family but own opposite effects in regulating the development of cancer like Let-7 family. Characterizing the underlying roles of those miRNAs may be propitious to HCC treatment. Recently, exosomes are found to have a large content of miRNAs, which brings a bright research prospect. In addition, the knowledge of the emerging functions of lncRNAs and circRNAs in drug resistance or other aspects in cancer is only the tip of the iceberg. The evidence of ncRNAs in clinical application is still insufficient. More clinical trials need to be further launched in the future. We believe that ncRNAs combine with chemotherapy will be an effective strategy for advanced liver cancer.

## Abbreviations

5-FU: 5-fluorouracil
ABC: ATP-binding cassette
ADM: adriamycin
circRNA: circular RNA
DHFR: dihydrofolate reductase
EMT: epithelial to mesenchymal transition
HBV: hepatitis B virus
HCC: hepatocellular carcinoma
IFN: interferon
LncRNA: long non-coding RNA
MDR: multidrug resistance
MRP: MDR-associated protein
ncRNA: non-coding RNA
Nrf2: NF-E2-related factor 2
P-gp: P-glycoprotein
Sirt1: silent information regulator 1
TP53INP1: tumor protein p53-induced nuclear protein 1
TUG1: taurine up-regulated gene 1
UTR: untranslated region
VCR: vincristine
wt-p53: wild-type p53
